# Using simulation models to investigate the cumulative effects of sowing rate, sowing date and cultivar choice on weed competition

**DOI:** 10.1016/j.cropro.2016.05.002

**Published:** 2017-05

**Authors:** Izzadora K.S. Andrew, Jonathan Storkey

**Affiliations:** Agro-Ecology Department, Rothamsted Research, Harpenden, Hertfordshire, AL5 2JQ, UK

**Keywords:** Competition, Cultural weed control, INTERCOM, Suppression, Tolerance

## Abstract

With the increasing pressure on crop production from the evolution of herbicide resistance, farmers are increasingly adopting Integrated Weed Management (IWM) strategies to augment their weed control. These include measures to increase the competitiveness of the crop canopy such as increased sowing rate and the use of more competitive cultivars. While there are data on the relative impact of these non-chemical weed control methods assessed in isolation, there is uncertainty about their combined contribution, which may be hindering their adoption. In this article, the INTERCOM simulation model of crop/weed competition was used to examine the combined impact of crop density, sowing date and cultivar choice on the outcomes of competition between wheat (*Triticum aestivum*) and *Alopecurus myosuroides*. *Alopecurus myosuroides* is a problematic weed of cereal crops in North-Western Europe and the primary target for IWM in the UK because it has evolved resistance to a range of herbicides. The model was parameterised for two cultivars with contrasting competitive ability, and simulations run across 10 years at different crop densities and two sowing dates. The results suggest that sowing date, sowing density and cultivar choice largely work in a complementary fashion, allowing enhanced competitive ability against weeds when used in combination. However, the relative benefit of choosing a more competitive cultivar decreases at later sowing dates and higher crop densities. Modeling approaches could be further employed to examine the effectiveness of IWM, reducing the need for more expensive and cumbersome long-term *in situ* experimentation.

## Highlights

• Modeling weed control strategies reduces the need for long-term experimentation.
• The INTERCOM model can be used to investigate Integrated Weed Management.
• Combining cultivar, sowing density and dates is effective against *Alopecurus myosuroides*.
• A combination approach reduces yield loss and enhances weed control.

## 1 Introduction

In agricultural systems, a careful balance is required between producing a high value crop yield and minimising costs. In this regard, weeds are the most serious potential threat to maintaining profitable farming systems, responsible for inflicting approximately 34% potential yield loss globally (Oerke, 2006). The introduction of herbicides in the 1960s allowed effective and relatively cheap control of weed species. Unfortunately, over-reliance on herbicides has led to widespread resistance in many problematic weed species (Heap, 1997, Moss et al., 2011) and the current herbicide-based weed control paradigm is widely considered to be unsustainable. In response, an approach which combines herbicides with a range of non-chemical (or ‘cultural’) weed management options, termed Integrated Weed Management (IWM), is increasingly being employed to compensate for loss of herbicide efficacy (Bond and Grundy, 2001, Lutman et al., 2013, Andrew et al., 2015).

Non-chemical control techniques employed in IWM are numerous and can be divided into those implemented over several seasons, including rotational ploughing and increased crop diversity, and within-season measures. The latter include increased sowing rate and growing more competitive cultivars to minimise weed seed return. Within-season options, that aim to shift the competitive balance in favour of the crop, are the focus of this paper. In most systems, non-chemical weed management options will be employed in combination with herbicides but by increasing crop competitiveness, the required efficacy and reliance on herbicide control is reduced. In the UK, non-chemical techniques are increasingly being utilised to enhance control of the weed species *Alopecurus myosuroides* Huds. in winter wheat (*Triticum aestivum* L). This annual grass species can cause substantial losses to wheat (Storkey et al., 2003) and herbicide resistance is widespread in North-West Europe (Moss et al., 2011, Lutman et al., 2013, Keshtkar et al., 2015), and is the focus of this study.

Non-chemical control tools require financial or temporal investments and their effectiveness varies from year to year. The resulting uncertainty means non-chemical control strategies tend only to be utilised when herbicides begin to fail (Bastiaans et al., 2008), as is currently the case for the control of *A. myosuroides* in the UK. Recommended non-chemical control options for *A. myosuroides* in the UK include rotational ploughing, use of spring crops (*A. myosuroides* mainly germinates in the autumn), delayed sowing date (to allow the use of a stale seedbed), increased crop sowing rate and the use of more competitive crop cultivars (Lutman et al., 2013).

Non-chemical control techniques are infrequently studied in combination, owing to the scale of experiment required, and data are therefore lacking on whether combined effects are additive, synergistic or antagonistic. Weed control measures have previously been examined with the use of simulation models. Models allow a means of studying scenarios *in silico*, providing insight without the need for large-scale experimentation. One well developed and validated model of crop/weed competition is INTERCOM, initially developed by Kropff and Spitters (1992) which has been parameterised for several crop and weed species since its inception (van Ittersum et al., 2003). When tested using sugar beet and *Chenopodium album* L., the original model explained 98% of the variation in yield loss (Kropff et al., 1992) and since then has been adapted to model competition from a range of weed species, including *A. myosuroides* in winter wheat under UK conditions (Storkey and Cussans, 2007). The model includes a range of eco-physiological parameters that determine the competitive balance between crops and different weed species and is weather driven allowing variability in output owing to environmental stochasticity to be quantified. The model can be used to examine the impact of sowing density, sowing date and crop cultivar on the outcome of crop/weed competition.

In this paper, we demonstrate how the INTERCOM model of plant competition can be utilised to observe the combined effect of sowing density, sowing date and cultivar choice, using wheat and *A. myosuroides* as model species. Furthermore, we discuss the advantages and disadvantages in employing models to understanding weed control initiatives and advising on their future use to support the implementation of IWM.

## 2 Materials and methods

### 2.1 Description of the INTERCOM model

The INTERCOM model makes predictions of the outcomes of competition between a crop and a weed based on leaf area production and distribution through the canopy in daily time steps (Kropff and van Laar, 1993). The primary driving environmental variables are photoperiod, temperature and available water. Temperature and water are growth-limiting, whilst accumulated photoperiod and thermal time mediate switches between developmental stages. The model has three discrete periods. Before plants begin competing for resources, growth is sink limited and modelled using an exponential relationship with biological time. In the original model, thermal time was used but, in later versions, a variable incorporating incident radiation (effective day degrees) was found to better capture differences between the growth of autumn and spring emerging cohorts (Storkey, 2004). A total green area index (GAI) of 0.75 is used as a switch between sink and source limiting growth – the next phase of the model. The ability of crop and weed to intercept light is determined through their share of the canopy (leaf area index), leaf traits related to light absorption (such as specific leaf area) and the vertical distribution of leaf area through the canopy. The model also accounts for changes in leaf traits and light absorption over time (Storkey, 2005). Plant height growth is predicted to follow the logistic function against accumulated photothermal time, as defined by Spitters (1989). Precipitation data and soil water balance functions are included in the model, using calculated rates of transpiration and evaporation. Water becomes limiting when soil moisture falls below a pre-determined level, and the relationship between the potential growth rate and water limited growth determined from an empirically derived relationship The final phase of the model is senescence and, for wheat, grain filling. Re-allocation of resource from stems and leaves to grain is modelled using functions from the Sirius model of wheat growth (Jamieson et al., 1998).

The version of INTERCOM utilised in this study has been parameterised for winter-sown wheat and *A. myosuroides* for improved description of winter wheat growth and partitioning (see Storkey and Cussans, 2007, where a detailed description of the model can be found). It was amended for the purposes of this study in C++ as described below.

### 2.2 Parameterising INTERCOM for wheat cultivars

In the winter wheat/*A. myosuroides* model, wheat was originally parameterised using data from the cultivar Consort (Storkey and Cussans, 2007). However, it has been frequently demonstrated that wheat cultivars differ in their ability to compete against weeds. While INTERCOM has been used in the past to inform the breeding of competitive rice cultivars (Bastiaans et al., 1997), here, we take the novel approach of using the model to quantify the relative impact of cultivar choice on weed competition in the context of variable sowing rate and sowing date. The variability in cultivar competitive ability has been attributed to numerous plant traits, including height, leaf area and developmental speed (Andrew et al., 2015). Many of these are traits utilised by INTERCOM to make predictions of competitive outcomes.

**Fig. 1:**
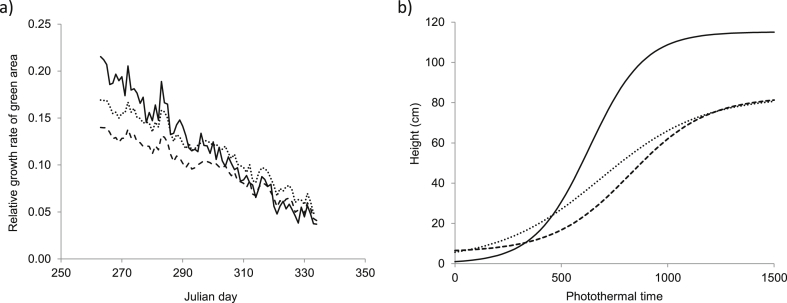
Differences between two contrasting cultivars used in the *in silico* experiments for two traits: a) relative growth rate of green area (cm^2^ cm^−2^ day^−1^) calculated using the daily mean temperature averaged over ten years and b) increase in plant height calculated using photothermal time, (- - -) KWS Santiago, (…) Duxford and (^___^) *A. myosuroides*.

The model was parameterised for two contrasting wheat cultivars, Duxford and KWS Santiago. These cultivars were selected based on three years of study (2012, 2013, 2014) in outdoors containers, where they represented the extremes in terms of competitiveness when compared to a range of ten modern wheat cultivars. Duxford was frequently reported as the strongest suppressor of *A. myosuroides* across three years of study, whilst KWS Santiago was frequently the poorest performer (Andrew, 2016). Using data collected from a series of outdoor, container-based experiments based at Rothamsted Research, UK, data were available to parameterise the model for different cultivars. To parameterise seedling growth rate, the protocol used in Storkey (2004) was followed; sequentially sampling seedlings over a two month period. For parameters determining resource competition, the cultivars were grown in competition with *A. myosuroides* in outdoor containers (40 × 32 cm) in a fully replicated experimental design repeated over three years and a range of morphological traits measured through the season. A selection of the original model parameters for wheat (cv. Consort) and for the two contrasting cultivars can be found in Table 1. The model was separately parameterised for each cultivar in C++. The main differences between the cultivars were in their rate of development, early height and early vigour (Fig. 1). Duxford tended to have a relatively erect canopy structure early on and a high seedling growth rate (related to a higher specific leaf area and lower partitioning to roots) whereas KWS Santiago tended to delay shoot extension and be relatively prostrate in the seedling stage.

### 2.3 Simulations

A number of *in silico* experiments were done using INTERCOM. Firstly, data input for INTERCOM can be amended to reflect the density of wheat and *A. myosuroides* in the stand and wheat sowing date; the interaction of these two factors was analysed using the original parameters for the cultivar, Consort. Crop densities between 100 and 400 wheat plants m^−2^ were selected to represent the potential to increase the competitive ability of the wheat canopy with *A. myosuroides* without changing cultivar choice. A range of sowing dates was chosen to reflect a realistic period for sowing winter wheat in the UK (15 September – 14 November). Emergence times after sowing were kept constant at seven days for *A. myosuroides* and 10 days for wheat, and the *A. myosuroides* density was maintained at 80 plants m^−2^ across all simulations. To quantify the interaction of sowing date and sowing rate on crop canopy competitiveness, the model was run using 49 combinations of rate × date, using intervals of 50 plants m^−2^ for crop density and 10 days for sowing date. The simulation model was run using radiation, temperature and precipitation data recorded at Rothamsted meteorological station for harvest years 2005–2014, providing yearly predictions of percentage crop yield loss and *A. myosuroides* above-ground dry weight (m^−2^).

The second experiment analysed the differences between the cultivars, Duxford and KWS Santiago, at a range of crop densities (this time increased to a maximum of 600 plants m^−2^) and a similar range of sowing dates as the first experiment. The ten years of weather data were used and the mean and standard error for crop yield loss calculated for each combination of cultivar × crop density or cultivar × sowing date. Finally, the effect of variable weather was made the focus of a further analysis, using a small number of cultivar × sowing date × crop density scenarios. A preferred sowing date under weed-free scenarios (20 September) was chosen along with a later sowing date, utilised to reduce *A. myosuroides* competitive ability and its germination within the crop competitive ability (20 October) (Melander, 1995, Lutman et al., 2013). Two realistic crop densities were also chosen, 150 or 300 plants m^−2^. Each combination of sowing date and crop density was input into INTERCOM using the parameters for either Duxford or KWS Santiago using weather data from each of the ten years. We assumed that the yearly weather data are temporally independent which allowed the differences between the cultivars, sowing dates or drilling dates (and interactions between them) to be analysed in the context of this inter-annual environmental variability using ANOVA. All statistical analysis of data was conducted in Genstat 16 (VSN International, 2013).

## 3 Results

**Fig. 2:**
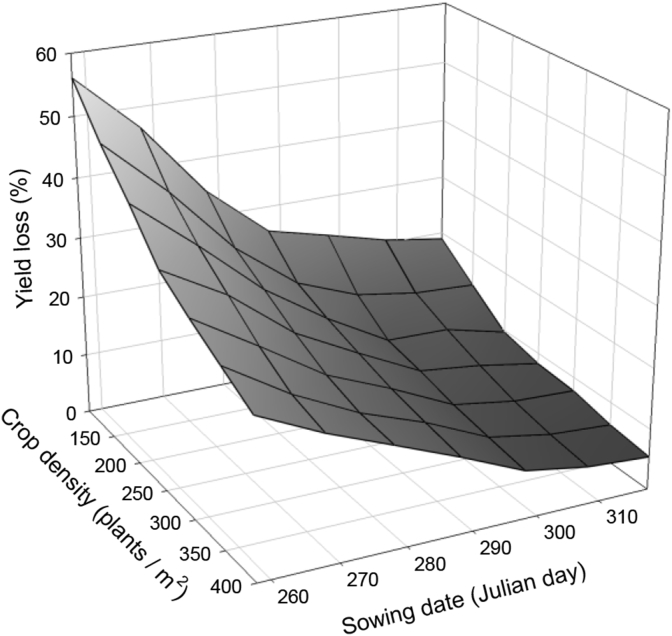
Interaction of crop density (100–400 plants m^−2^) and sowing date (15th September to 14th November) calculated as the mean output for each combination of density × date using weather data from 2005 to 2014. In all scenarios, a weed density of 80 plants m^−2^ was used and an emergence date for crop and weed of 7 and 10 days after sowing respectively.

In the model's predictions, percentage yield loss and *A. myosuroides* biomass at maturity were closely correlated (r = 0.86; P < 0.001). As such, although percentage yield loss is presented, the model predicted an equivalent reduction in weed biomass. In addition, the relationship between *A. myosuroides* biomass at maturity and seed production is observed to be positively correlated, allowing the output to be used to predict seed return under different scenarios. An increase of 10% yield loss was associated with approximately 15,000 additional weed seeds produced. When using parameters for a standard cultivar, Consort, the model predicted decreasing yield loss with both increasing crop density and a later sowing date; in both cases the relationship was non-linear (Fig. 2).

**Fig. 3:**
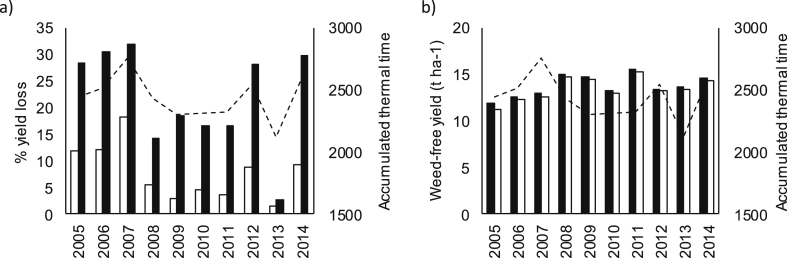
INTERCOM predictions using two contrasting cultivars showing impact of variable weather on a) percentage yield loss from years 2005–2014, and b) weed free wheat yield; the accumulated thermal time of each year is included as the dashed line. ■ = Duxford; □ = KWS Santiago. Crop density 300 plants m^−2^, sown 20 September, *A. myosuroides* density 80 plants m^−2^.

**Fig. 4:**
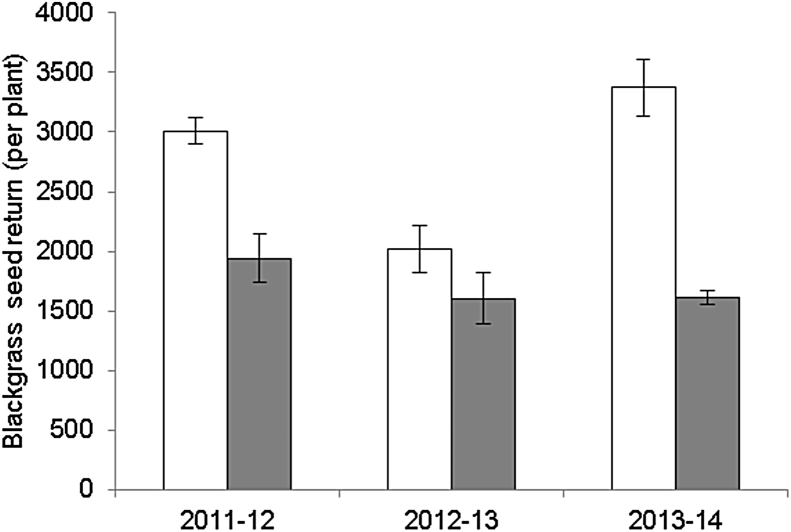
The seed return per plant of *A. myosuroides* (approx. 80 plants m^−2^ equiv.) when grown alongside one of two cultivars (275 plants m^−2^ equiv.) across three years in a container-based experiment.  = Duxford; □ = KWS Santiago. Mean temperature in 2011–12 was 8.3 °C, in 2012–13 was 6.3 °C and in 2013–14 was 8.9 °C.

Higher yield loss was always observed for KWS Santiago (F_1,76_ = 34.33, P < 0.001), regardless of crop density or sowing date. However, yield loss varied for both cultivars across the different seasons and the relative difference between the cultivars was highly weather dependent (Fig. 3a). These predictions are in line with empirical observations of weed suppression from the container experiments used to parameterise the model for Duxford and KWS Santiago (Fig. 4).

Accumulated thermal time was an important determinant of yield loss predictions in the INTERCOM model for both cultivars, with lower temperatures resulting in decreased yield loss (F_1,76_ = 21.62, P < 0.001) and reduced differences between the cultivars. In the coldest year, 2013, the model reported the lowest yield loss prediction of 3.2%, whilst the second highest was in the warmest year (2006), with 17% yield loss. The predicted weed-free yield of wheat suffered no equivalent detriment in the colder years (Fig. 3b), implying that temperature has a stronger impact on *A. myosuroides* competitive performance.

Percentage yield loss at a crop density of 150 plants m^−2^ averaged 15%, decreasing to 9.4% when crop density was increased to 300 plants m^−2^ (P = 0.01; 1 d.f.). Delaying sowing by 30 days also reduced percentage yield loss, with 19.1% yield loss on 20 September sowing dates and 5.3% yield loss when the crop was sown on 20 October (P < 0.001; 1 d.f.) (Table 2).

The INTERCOM model predicts that Duxford is the most competitive cultivar across all simulation years, with KWS Santiago suffering 18.5% yield loss whilst Duxford only suffered 5.89% yield loss (P < 0.001; 1 d.f.) (Table 2). There was no significant interaction between sowing date, sowing density and cultivar choice, suggesting they behave cumulatively when employed together to reduce percentage yield loss.

The effects of changing crop cultivar, sowing density and sowing date on weed-free yield was restricted only to sowing date, with delayed sowing resulting in a mean decrease in yield from 13.56 t ha^−1^ to 12.79 t ha^−1^ (P < 0.002; d.f. 1) (Table 3).

**Fig. 5:**
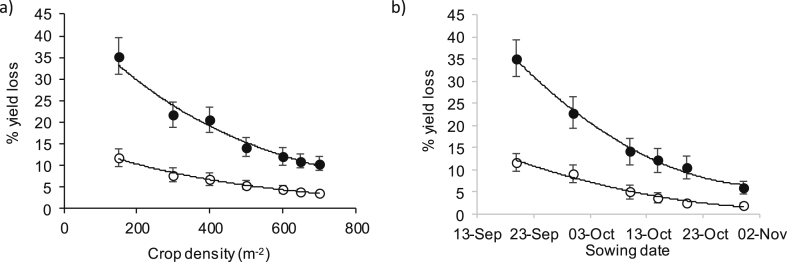
The predicted percentage yield loss for () Duxford and (●) KWS Santiago when sown at a) different densities (with a sowing date of 20 September) and b) different sowing dates (with a crop density of 150 plants m^−2^). In both cases, weed density was 80 plants m^−2^ and dates of emergence were 10 and 7 days after sowing for the crop and weed respectively.

The model anticipates Duxford to outperform KWS Santiago at all densities, and the benefit of increased sowing density reduces with each subsequent increase (Fig. 5a). In order for KWS Santiago to achieve a similar yield loss to Duxford when sown at 150 plants m^−2^ (mean percentage yield loss of 11.7), its stand density must be increased to 640 plants m^−2^ (Fig. 5a). A similar effect is observed with sowing date, with Duxford consistently more competitive than KWS Santiago and the benefit of delayed sowing is reduced with each additional day (Fig. 5b). In order for KWS Santiago to achieve a similar yield loss as Duxford sown at 150 plants m^−2^ on 20 September, it must be sown on 16 October. However, as sowing density increased or sowing date was delayed, the relative benefit of using a competitive cultivar decreased.

## 4 Discussion

There are various non-chemical control strategies available to farmers, and these are often utilised in IWM. However, there is a need to understand how they perform in combination and how they interact with variable weather in order to maximise weed control and minimise yield loss (Barzman et al., 2015). The necessary field experiments to investigate this would require a scale (temporal and spatial) that would make them difficult to conduct and complicated to analyse. The use of simulation models such as INTERCOM can provide valuable insight into their combined effect on crop-weed competitive interactions (van Ittersum et al., 2003).

The predictions of the reduction in yield loss with increasing crop density were in agreement with the published literature (Mennan and Zandstra, 2005). The model predicted an average reduction in seed production of 25% when crop density was increased from 100 to 300 plant m^−2^. This is an equivalent increase in crop competitiveness as was reported in Lutman et al. (2013) where these treatments resulted in reductions in *A. myosuroides* head density of approximately 32%.

A similar comparison cannot be made with data from Lutman et al. (2013) on the impact of delayed sowing on weed competition as we did not incorporate the effect of reduced weed establishment at late sowing dates. This would be a useful improvement of the models. However, the model output was realistic in that it predicted that in the wheat – *A. myosuroides* scenario, the crop acquires a competitive advantage when sown at higher densities and at later sowing dates. The benefit of increased sowing density has been observed in various crop-weed associations (Christensen et al., 1994, Melander, 1995, Cosser et al., 1997, Roberts et al., 2001, Lutman et al., 2013). However, we demonstrated an additional benefit of delayed sowing; the difference in relative growth rate between the crop and the weed is greatest at warmer temperatures, earlier in the sowing window. By delaying sowing, the competitive advantage of the weed is reduced. This finding would be welcomed by those seeking a boost to their weed control by delaying sowing wheat in the fields with the worst weed problems.

The maximum reduction in *A. myosuroides* head density caused by cultivar differences was reported as 52%, with a mean across multiple experiments of 30% (Lutman et al., 2013). This compares to the current study with a maximum difference in *A. myosuroides* biomass and, therefore, seed production between the two cultivars in a given year of up to 80%. This may be because there are facets of competition that the model does not capture. Below-ground competition is estimated based on the proportional share of root space between the competing species, which may not provide an accurate representation of acquisition of limited soil resources. *In situ* validation of the model's predictions of the combined impact of cultivar choice, crop density and sowing date would be of value (Deen et al., 2003). It is possible that there is a trade-off between early vigour (where Duxford ‘wins’) and later season competition for below-ground resources which would have the effect of reducing the differences between the cultivars. Due to the lack of data on rooting characteristics, when assessing cultivar differences, the model is weighted towards above-ground early growth traits. Because of this, the predictions of absolute differences need to be treated with caution. However, it is likely that the pattern of the interaction with sowing rate and sowing date are more robust.

The model suggests that cultivar choice is a viable, low-risk alternative in weed management. Cultivars are observed to differ in competitive ability in field studies (Christensen et al., 1994, Lemerle et al., 1996, Wicks et al., 2004), and to work in combination with sowing density (Mennan and Zandstra, 2005). Studies have reported a lack of consistency in the ranking of cultivars in studies comprising of multiple years (Vandeleur and Gill, 2004). In addition, the degree of weed control and tolerance to weed competition is observed to vary between years. This degree of uncertainty is reflected in the model and attributed to lower temperatures, perhaps compromising the ability of *A. myosuroides* to compete (Melander, 1995).

The use of a competitive cultivar has an additive affect, suggesting that similar cultivars may be employed in combination with later sowing and higher crop densities to enhance weed control. Many farmers are familiar with the benefits of delaying sowing and increasing sowing density in order to control *A. myosuroides*, but uptake can be restricted when farmers are less certain of their outcomes (Lutman et al., 2013). The competitive ability of modern cultivars is less understood, and its understanding is confounded by their short commercial lifespan within UK agriculture (Andrew et al., 2015). In order for farmers to utilise this tool, they need to know the additional benefit a competitive cultivar would confer. It is proposed that this is best communicated in reference to other weed control strategies. For example, INTERCOM predicts that, in order for KWS Santiago to reduce yield loss to the same extent as Duxford at 150 plants m^−2^, it must be sown at over 600 plants m^−2^. Such a high density of wheat is an unrealistic target for producers due to increased risk of lodging and the cost of the additional seed, making Duxford a viable alternative to increase crop competitive ability.

The same principle applies to sowing date. In order for KWS Santiago to match Duxford's lower percentage yield loss when sown on 20 September, an approximate sowing date of 16 October is advised by the model. Delayed sowing has associated risks not captured by INTERCOM, such as poor crop establishment or poor weather in late autumn preventing the farmer from sowing the crop at all. Although maximal benefit is achieved by delaying until early November, few growers are willing to risk a late sowing date (Lutman et al., 2013). Selecting Duxford over KWS Santiago would allow for the equivalent reduction without the risk.

An increase to crop density and sowing date follows the principle of diminishing returns, expressed as a rectangular hyperbola, which is accounted for by the model. For density, this is owing to the fact that each additional wheat plant added to the stand will increase crop canopy dominance by a smaller relative quantity and intraspecific competition becomes more important (Cousens, 1985). As such, the use of a more competitive cultivar would produce an additional benefit which cannot be acquired through increasing sowing density alone.

The INTERCOM model is one of the most widely-employed models of crop/weed competitive interactions, and has been parameterised and validated for use in numerous species combinations (Zimdahl, 2004). Here, we have used the model to demonstrate its utility in predicting the behaviour of a specific crop/weed combination of immediate relevance to European cereal production. However, there is the potential to take a similar approach to study systems with alternative or multiple weed species (Storkey and Cussans, 2007) to ask questions such as ‘are the differences in weed suppression between cultivars similar when competing with different weeds’? In these scenarios, the model could provide enormous insight into the combined benefit of non-chemical control options and reduce the need for large, complex experiments. It's flexibility in adjusting for growth rates, density and sowing date allow it to examine crop canopy competition under different climatic conditions, and it is readily adaptable to suit the crop/weed scenario of interest where light availability is a crucial component in determining the outcomes of competition. A more detailed understanding of below-ground competition may be required to increase the robustness of the predictions when water or nutrients are limiting.

## 5 Conclusions

The INTERCOM model for wheat – *A. myosuroides* simulates IWM on final competitive outcomes as would be largely expected from the literature, and implies that delayed sowing date, increased crop density and competitive cultivars work well in combination. Sowing a cultivar more similar to Duxford than to KWS Santiago could provide enhanced *A. myosuroides* suppression and yield retention without the risks inherent to sowing date and crop density. This approach, if applied to other crop-weed combinations, could provide valuable information on IWM measures, reducing the need for repeated, expensive and long-term experimentation and help growers to make better informed weed management decisions.

## Figures and Tables

**Table 1:** Parameter values for the INTERCOM model. Values for cultivar Consort are those included in the original version of the model developed for winter wheat (Storkey and Cussans, 2007). Cultivar values are those used to parameterise for respective cultivar. RWR = root weight ratio, SSA = specific stem area, SLA = specific leaf area, RGR_GA_ = relative growth rate of green area, L_0_ = initial green area, *a* = initial height, *c* = height asymptote, *b* = maximum growth rate, *m* = time of the point of inflexion (just prior to achieving the asymptote).

Trait	Consort (Storkey and Cussans, 2007)	Duxford	KWS santiago
RWR	0.71	0.705	0.681
SSA (m^2^ g^−1^)	0.003	0.00545	0.00504
Phyllochron (dd leaf^−1^)	90	67.5	69.5
SLA (m^2^ g^−1^)	0.019	0.0385	0.0346
RGR_GA_ (cm^−2^ cm^−2^ tt^−1^)	0.0089	0.0116	0.0096
L_0_ (cm)	0.64	0.674	0.715
*Logistic functions for height*			
*a* (cm)	7.4	1.36	5.73
*c* (cm)	77.9	81.845	77.299
*b* (cm ptt^−1^)	0.0085	0.004218	0.005559
m (ptt)	624	685.0	822.6

**Table 2:** The percentage yield loss predicted by INTERCOM for wheat cultivars Duxford and KWS Santiago under different crop density and sowing date combinations. ± indicates standard error.

Cultivar	Density (plants m^−2^)	20 September		20 October	
		Sowing date			
Duxford	300	7.8	±0.524	1.6	±0.119
	150	11.7	±0.613	2.5	±0.206
KWS Santiago	300	21.7	±0.953	6.6	±0.441
	150	35.3	±1.327	10.5	±0.789

**Table 3:** The weed-free yield (t ha^−1^) predicted by INTERCOM for wheat cultivars Duxford and KWS Santiago under different crop density and sowing date combinations. ± indicates standard error.

Cultivar	Density (plants m^−2^)	20 September		20 October	
		Sowing date			
Duxford	300	13.8	±0.118	12.9	±0.088
	150	13.7	±0.122	12.9	±0.087
KWS Santiago	300	13.4	±0.126	12.7	±0.087
	150	13.4	±0.135	12.7	±0.086
